# The effect of methenamine on vascular development: Experimental investigation using in vivo and insilico methods

**DOI:** 10.18502/ijrm.v13i8.7497

**Published:** 2020-08-19

**Authors:** Hadi Tavakkoli, Masoud Imani, Mohammad Rahchamani Seyyed, Mohsen Rezvani

**Affiliations:** ^1^Department of Clinical Sciences, Faculty of Veterinary Medicine, Shahid Bahonar University of Kerman, Kerman, Iran.; ^2^Department of Veterinary Medicine, Shahid Bahonar University of Kerman, Kerman, Iran.

**Keywords:** Methenamine, Angiogenesis modulating agents, Vascular endothelial growth factor A, Extraembryonic membranes.

## Abstract

**Background:**

Methenamine is a worldwide antibacterial agent for urinary system infections in human and animals. The effect of methenamine consumption during early phase of pregnancy is not fully clarified in previous studies. Vascular development is the essential part of the early embryonic growth.

**Objective:**

In this study, we used chicken chorioallantoic membrane to evaluate the effects of methenamine administration on angiogenesis process as a model.

**Materials and Methods:**

In this experimental study, 20 Ross 308 eggs (mean weight 55 ± 4) were incubated. The eggs were divided into two equal groups (n = 10/each). In the first group, methenamine (150 mg/kg egg weight) was injected on the shell membrane, and in the second group (control group) phosphate-buffered salineas injected. Methenamine was inoculated at 96 and 120 hrafter incubation; 24 hrafter the last inoculation, the eggs were removed and the egg's shell was incised. Then, the development of vascular network and vascular endothelial growth factor Aexpression was evaluated.

**Results:**

Angiogenesis was significantly decreased after methenamine treatment. The indexes such as areas containing vessels, the vessels' length, the percentage of angiogenesis developing areas, and vascular complexity in the treatment group receiving methenamine were significantly reduced compared to the control group. *Vascular endothelial growth factor A*expression was suppressed in the methenamine treated group.

**Conclusion:**

According to the achieved results, it was defined that methenamine could have an inhibitory effect on the growth and development procedures of extraembryonic vasculature.

## 1. Introduction

Methenamineis aheterocyclic organic compound with the formula (CH${}_{2}$)6N4 that has been widely used as a safe and relatively effective antibacterial for urinary tract infections. Methenamine, in acid pH of urine, releases ammonia and formic acid, and the formic acid is bactericidal (1). In case of chronic urinary infection, methenamine is a good choice for decreasing bacterial growth (lowering urine bacterial count) or for prophylaxis (keeping urine sterile after it has been cleared from bacterial infection by antibiotics) (2). Considerations that encourage the methenamineadministration include: i) its antibacterial effect in vitro, ii) the lack of effect on gut flora, iii) the apparent absence of bacterial resistance to low formaldehyde concentrations, iv) its relatively cheapness, and v) its reported low toxicity (3).

Production and proliferation of blood vessels are procedures necessary for the normal growth and development of tissue (4). After puberty, angiogenesis does not occur physiologically, except in the reproductive system of female, and in the placenta during pregnancy. The expansion of vascular networks during pregnancy or normal and pathological angiogenesis is related to the growth factors and interactions between cellular and extracellular matrix (5). During embryogenesis, development of blood vessels is done by two procedures including vasculogenesis and angiogenesis (6). Growth factors' roles in both procedures are well-clarified. Among these growth factors, the most two important factors are vascular endothelial growth factor (VEGF) and basic fibroblast growth factor (bFGF) that promote both embryonic vascular network initial development and new blood vessels formation during reproductive cycle of female, development, and wound healing (7). During pregnancy, vascularization of the human embryo takes place in the early stages, approximately the second week post conception and is appeared first in the extraembryonic areas (8). Vascularization of the chorionic villi is a cornerstone for embryonic development and that normal chorionic villous vascularization is essential for the undisturbed development of pregnancy (9).

Hydropic degeneration, fibrosis and lower level of vascular density have been detected in the cases of intrauterine embryonic death (10). Other illnesses and syndromes such as pre-eclampsia, intrauterine growth restriction, gestational diabetes and maternal diabetes mellitus, which disrupt the process of angiogenesis, can also damage the developing fetus (11). In the case of thalidomide toxicity, it has been reported that all congenital defects could be due to antiangiogenic action of thalidomide during embryogenesis (12). Therefore, any drug that has an effect on the angiogenesis process will most likely affect the development of the fetus during pregnancy. There are a few studies on methenamine toxicity during pregnancy in literature. In the FDA grade of drug consumption during pregnancy, the grade of methenamine is C, which means that there is no standard trial to prove the safety of methenamine during pregnancy (13). The chick embryo chorioallantoic membrane (CAM) is an extraembryonic membrane that is commonly used in vivo to study angiogenic or antiangiogenic substances (14).

To the best of our knowledge, there is no investigation on angiogenic/antiangiogenic properties of methenamine. In this study, we tried to study the angiogenic/antiangiogenic properties of methamine in order to get informed about its possible side effects during pregnancy. We also identified the possible interaction of methenamine with VEGF-A protein by computer-aided simulation.

## 2. Materials and Methods

This section explained in terms of A) effect of methenamine extract on the early embryonic angiogenesis, B) effect of methenamine extract on the expression of VEGF-A, and C) assessment of methenaminebinding to VEGF-Awith the help of a computer program.

### Effect of methenamine on early embryonic angiogenesis

Angiogenesis in the early embryonic phase and the effect of methenamine on this process was evaluated by the evaluation of vascularization on the chick's extraembryonic membrane (EEM). The procedure is explained as follows.

#### Eggs

Fertile chicken eggs (Ross 308) with the average egg-weight of 52.5 ± 0.6 gr were obtained from the Mahan Breeder Company, Kerman, Iran. In this farm, the condition to rear breeder birds was standard.

#### Embryo treatment and image acquisition

The eggs were kept in vertical position in an electrical incubator (Belderchin Damavand Co. PLC-DQSH, Tehran, Iran) at a temperature of 37.5°C and relative humidity of 60%. During all procedure, 70% ethanol solution was used for the disinfection of equipment that were in contact with the eggs. On the fourth day (or: on day 4) of incubation, an orifice with diameter of approximately 2 mm in the eggshell at the site of the air space was made. An injection of 50 μl of either methenamine (150 mg/kg) or sterile phosphate-buffered saline as placebo was done in the created site on eggshell as a single drop on the inner shell membrane. The inner shell membrane was a permeable membrane and allowed passing of drugs into the underside membrane. Therefore, we inoculated the drug on the inner shell membrane. This method of treatment has beenused in various studies (15, 16). Ten eggs were assigned in each treatment. The eggs were re-treated at 24 hr after the first one (96 and 120 hr, days 4 and 5, of incubation period).

The treated dosage of the methenamine was selected following a preliminary study. In that study, we used various dosages of methenamine for treatment. The minimum gross abnormality and relatively higher survival rate were observed at the dose of 150 mg; hence the optimum treatment dosage of methenamine was selected as 150 mg for further experiments.

After injection, the orifice was closured by warmed paraffin (Merck, Darmstadt, Germany). This procedure has beenpreviously invented (17). On day 6 of incubation period, 4 ml of albumin was extracted from the pointed end of the eggs, using 5ml syringe with a 21-gauge needle, to allow the embryo to take some distance from the eggshell. The eggshell was cut somehow to create a window with a dimension of 2.5 cm to imagine the surface of EEM using stereomicroscope (Luxeo 4D Stereozoom Microscope, Labomed, CA, USA) attached to Canon SX200 camera supported by Luxeo software. High resolution images (4000 × 3000 pixels) were prepared with these equipment and saved as *.tif files using a 14.5-inch PC (Intel Core i3-390M, 2.66 GHz).

#### Vascular branching pattern analysis

**Figure 1:**
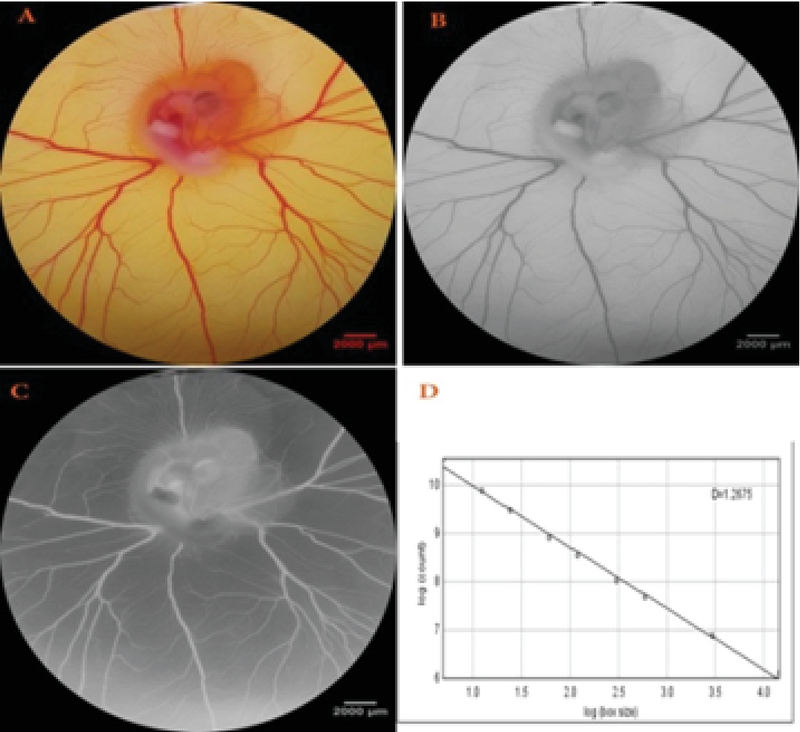
Calculation method of the fractal dimension (Df) value from the captured images. The oval is located right before the first main branch of the right lateral vitelline vein and has included a size of 177 mm${}^{2}$ (A). The extracted areas were converted into 8-bit format and processed to increase edge detection (B). Contrast was reversed for better detection of vessels(C). The Df is measured from the slope of the regression line using the box-counting method (D).

Software including Digimizer
Ⓡ
 4.3.0 (MedCalc Software, Mariakerke, Belgium), ImageJ
Ⓡ
 1.48 (National Institutes of Health, Bethesda, Maryland, USA) and MATLAB
Ⓡ
 (MathworksMatlab R2015a) were used to analyze the captured images from surface of EEM. For this computerized evaluation, a certain part of the microscopic image was selected. The selected part included an area with the size of 315 mm${}^{2}$ and 2987 × 2987pixels located at the right-lateralaspect ofvitelline vascular plexus. To detect the schematic template of the vascular network, images were converted to 8-bit format and then analyzed. Ultimately, the color scale of images was decreased to black and white format and changed into skeletonized picture. In skeletonized image, structural figure of object was seen. Indices including vessels area, total vessels length, and fractal dimension (Df) value werethe representative of vascular branching pattern (18) and were estimated by analysis of processed images. The Df was estimated with a box counting method on the processed images as previously described (Figure 1) (15).

#### Morphometric analysis of capillary density

Briefly, the contrast raised in the defined part, inside the right-lateral vitelline plexus that was previously selected. The effort was made to choose the steady parts in each case to desist subjectivity in analysis. The format of selected parts changed to black and white. The analysis was done on the areas with no vessels. The percentage of the areas comprising black pixels was quantified in five areas per case. These black pixels were the representatives of red color, or blood, in the original images. The mean of all areas calculated in each case was declared as the mean capillary area (MCA) (19).

### Effect of methenamine on the expression of VEGF-A

qPCR (quantitative real-time PCR) was used to determine the relative expression degree of VEGF-A gene in the chicken EEM. The extraction of total RNA from the EEM samples was done usingRNeasy
Ⓡ
 mini kit (Qiagen, Chatsworth, CA) based on the manufacture instructions (four samples per group). The concentration of RNA (ng) and its purity (260 nm: 280 nm) were assessed by spectrophotometric method via NanoDrop ND-1000 (NanoDrop ND-1000, Thermo Scientific, Wilmington, DE, USA). TaKaRa Prime Script${}^{\mathrm{TM}}$ RT reagent kits (Takara Bio, Inc., Shiga, Japan) was used forthe synthesis of cDNA and the procedure was done at 37°C for 15 min on 500 ng of the total RNA sample. Duplicate qPCR reaction was accomplished for each sample in Rotorgene Cycler system (Rotorgene 3000 cycler system, Corbett Research, Sydney, Australia) using a SYBR Green assay (SYBR Premix Ex Taq${}^{\mathrm{TM}}$ II, Takara Bio, Inc., Shiga, Japan) based on the recommended method. The specific sequences of reference gene and primers are presented in Table I (15). 86 bp segment of the VEGF-A mRNA genes were amplified by the primers. For the qPCR reaction,the following steps were followed: 1) holdingh treatment at 95°C for 1 min, 2) forty cycles included 10 sec at 95°C for denaturation, 15 sec at 60°C for primer annealing, and 20 sec at 72°C for extension. Expression levels were estimated relative to the expression levels of the chosen reference gene.

#### Molecular modeling

To evaluate the probable interactions between the methenamine and VEGF, a bioinformatic analysis was performed (20). The ligand structure was acquired from the PubChem database (PubChem CID: 4101) and was optimized by using Discovery Studio 2017 (DS 2017) software. The sequence of VEGF-A (*Gallus gallus*, Gene ID: 395909) was downloaded from the NCBI server, and conformational structure of human VEGF-A receptor-binding site was downloded from the RCSB server (PDB code: 4KZN). The structure of VEGF-A (4KZN) consist of a ligands. Tree dimensional structure of VEGF-A (*Gallus gallus*) was designed by SWISS-MODEL server. Partial charges and hydrogen were add to the protein and ligandand docking study was done to evaluate the binding affinity of methenamine to VEGF-A via Auto DockVina. The lowest energy ctructure was choosed for further analysis.

**Table 1:** The specific primers and reference gene sequences for quantitative real-time RT-PCR


**Gene (** ***Gallus gallus*** **)**		**Sequence (5'-3')**	**Product size (bp)**
**VEGF-A**			
	**Forward**	CAATTGAGA CCC TGGTGG AC	86
	**Reverse**	TCT CAT CAG AGGCACACAGG	
**GAPDH**			
	**Forward**	CCTCTCTGGCAAAGTCCAAG	176
	**Reverse**	GGTCACGCTCCTGGAAGA TA	

### Ethical consideration

All investigations were conducted in accordance with the Guiding Principles for the Care and Use of Research Animals and were approved by the ethics committee of the Shahid Bahonar University (code: IR.UK.VETMED.REC.1398.004).

### Statistical analysis

Statistical analysis was performed using the SPSS version 20 (Statistical Package for the Social Sciences, SPSS Inc., Chicago, IL, USA). One-way analysis of variance followed by Tukey's test was appliedto assess the significance of differencesin the vascular parameters and gene expression. A p-value of <0.05 was considered statistically significant.

## 3. Results

**Figure 2:**
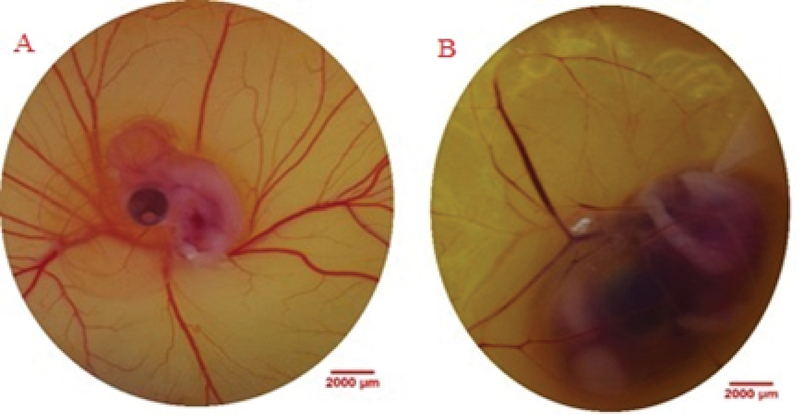
Embryonated eggs were administered at 96 and120 hrafterthe incubation. (A) Control embryo with normal extra-embryonic membrane vasculature is seen. (B) Embryonated egg received methenamineat the dosage of 150 mg/kg eggweight. Vascular disruption is demonstrated by decreased branching.

On day 6 of incubation, when the egg shell window was created for taking photos, Hamburger-Hamilton developmental stages of embryos were at 29. In the control group, a fine network of vitelline vessels surrounded the embryo (Figure 2a). In the methenamine group, an abnormal template of EEM vascular network was seen in embryos. Vascular disturbance was observed by a very obvious decreased branching (Figure 2b). The analysis of vascular branching pattern following methenaminetreatment is presented in Table II. Methenamine in dosage of 150 mg/kg alters the vascular branching pattern. Methenamine decreasedthe Df value of the vascular network in comparison with the controls (control, 1.338 ± 0.012; and 150 mg/kg eggweight, 1.222 ± 0.020 (95% CI -0.165 to -0.066); p < 0.001).

**Figure 3:**
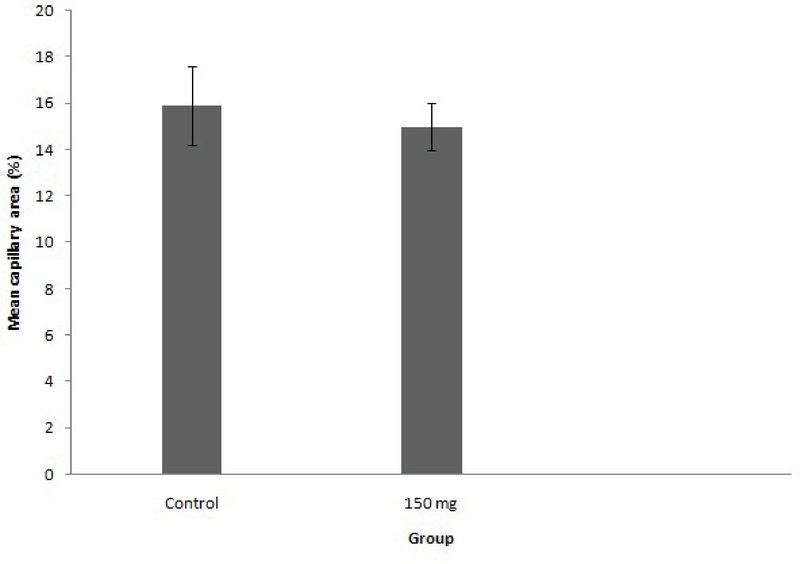
Calculation of mean capillary area (MCA) for the demonstration of the effect of methenamine on angiogenesis in CAM. Comparison was done between control (n = 10) and methenamine (n = 10)-treated group (error bars show standard error of mean; p < 0.05, One-way ANOVA).

Capillary density indicesaredemonstrated in Figure 3. MCA decreasedsignificantly in the vascular plexus of the treated embryos in group that receive methenamine (control, 15.59 ± 1.66; methenamine, 14.48 ± 1.01; p = 0.024.

### Expression of VEGF-A 

**Figure 4:**
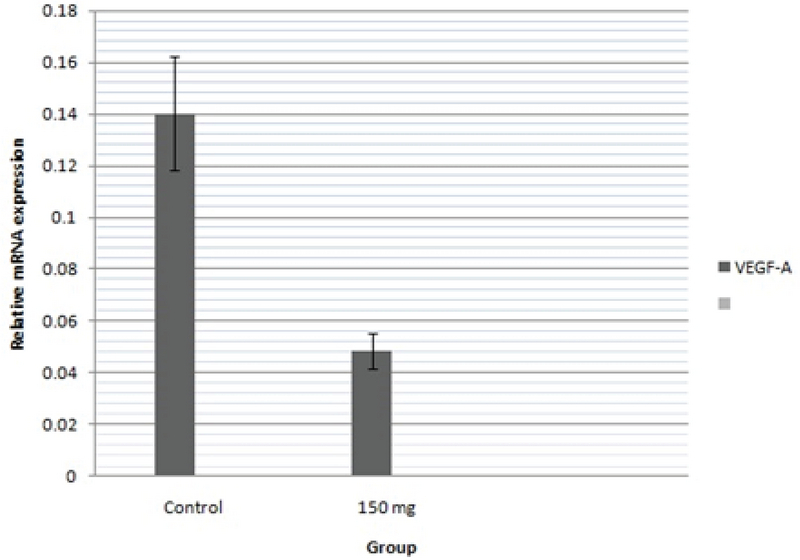
Relative expression of mRNA levels of *VEGF-A* gene in methenamine-treated group in comparison with control (error bars show standard error of mean; *p < 0.05, One-way ANOVA).

The relative expression of the VEGF-A gene was assessed using qPCR on day 6 of the incubation period. The relative mRNA expression levels of VEGF-A reduced in the methenamine-treated group compared to the control group (Figure 4).

### Methenamine bound to VEGF-A by molecular docking 

**Figure 5:**
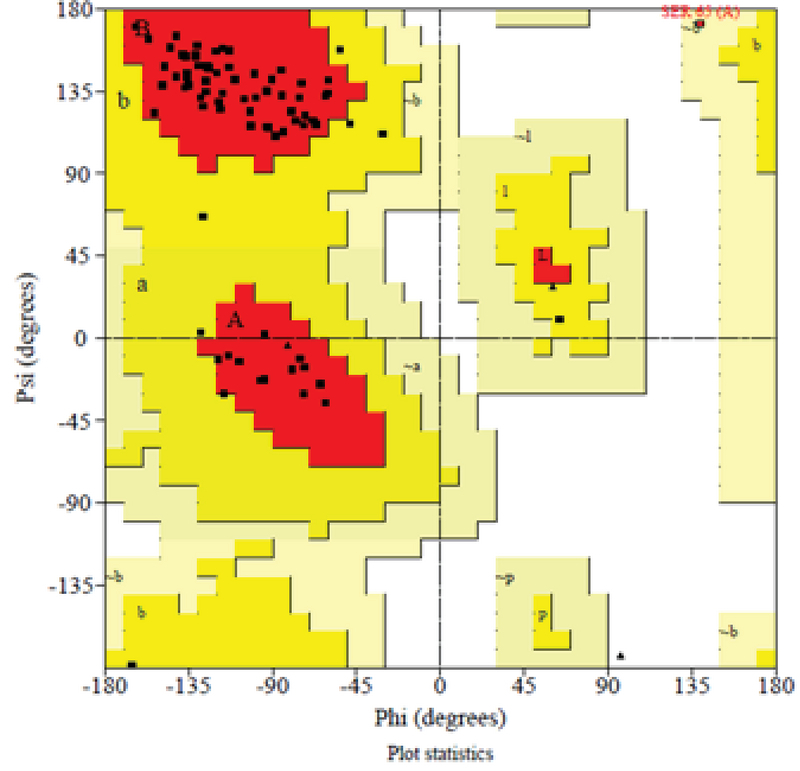
Ramachandran plot for protein model of VEGF-A (*Gallus gallus*). PROCHECK source information for residues in most favored regions is 90.4% and confirms that a good-quality model would be expected.

The conformational structure of VEGF-A (*Gallus gallus*) was created by SWISS-MODEL server. The generated structure was assessed by analyzing PROCHECK srver and the Ramachandran plot is showed in Figure 5. PROCHECK data for residues in most favored regions was 90.4%, residues in additional allowed regions was 8.2%, residues in generously allowed regions was 1.4%, and residues in disallowed regions was 0.0%. These analysis showed that a good structures were generated. The conformational analysis of VEGF-A protein is as bellow: molecular weight: 9537.99, the number of amino acids: 83; total number of negatively charged residues (Asp + Glu): 12; total number of positively charged residues (Arg + Lys): 7.

**Figure 6:**
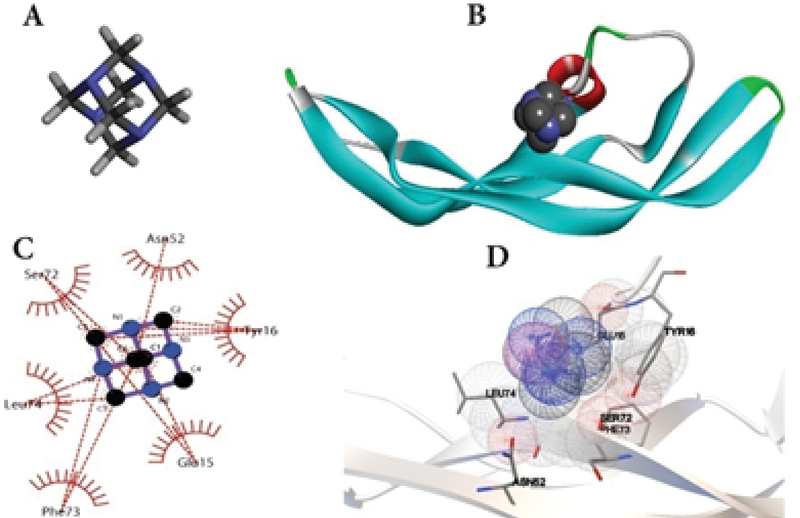
Binding mode of methenamine with VEGF-A (*Gallus gallus*). (A) 3D structure of original ligand. (B) Platform of methenamine inserted in the VEGF-Abinding site. (C and D) Hydrophobic and vandervase interaction between methenamine and amino acid residues of the nearby binding site.

The docking technique was performed to evaluate the binding affinity between protein and ligand. The acquired data confirmed that the active site of VEGF-A was docked with methenamine to make nine conformation. As showed in the binding structure (Figure 6), methenamine was bound to the active site of VEGF-A by hydrophobic and vandervase interaction with Ser72, Asn52, Tyr16, Glu15, Phe73, and Leu74.

**Table 2:** Vascular branching pattern analysis following methenaminetreatment


**Parameters**	**Group**	
	**Control**	**Methenamine (150 mg/kg)**
**Vessels area (%)**	30.22 ± 1.82${}^{a}$	24.73 ± 2.75${}^{b}$
**Total vessels length (Pixel)**	40.51${}^{a}$	28.91${}^{b}$
${}^{*}$Values are Mean ± standard error of mean;Values in a row followed by different superscripts differ significantly (p < 0.05)		

## 4. Discussion

Methenamine is a commonly used medicine for the treatment of urinary system infections. The most important concern about taking any medication is the side effects of drugs. Additionally, toxicity characteristic of drug during gestation is of great concern. Inthe present study, we studied the effect of methenamine administration on the early chicken embryo's angiogenesis as a model for drug consumption in early pregnancy. Methenamine at the dose of 150 mg/kg significantly reduced angiogenesis in extraembryonic chorioallantoic membrane.Andelman observed that the treatment of 531 women during pregnancy with methenaminehippurate had no signs of fetal injury (1). Undocumented surveillance study between 1985 and 1992 on the newbornsexposed to methenamine showed 3.8% malformation (21). Generally, the book of “drugs in pregnancy and lactation” emphasizes on the safety of methenamine in pregnant women. On the other hand, in this book, a collaborative perinatal projectisavailable that found an association between the consumption of drugs in pregnancy and fetal malformations (21).

In the present study, we found that methenamine reduced angiogenesis. To the best of our knowledge, there is no study in the literature about the antiangiogenic or angiogenic property of methenamine. On the other hand, angiogenesis disturbance during pregnancy could lead to embryonic or fetal growth abnormalities (10). After fertilization and entrance of fertilized ova into the uterus, there arethree important parts for the normal growth of embryo and maintenance of normal pregnancy in whichangiogenesis has a critical role to play: (1) uterus, (2) embryo or fetus, and (3) placenta. The endometrium, placenta, and decidua are rich sources of angiogenic growth factors and since the uterus and its enclosures require increased supply of blood during gestation, the production and development of new vessels occur (22, 23). Angiogenesis in embryo is indispensable for the formation of organs and their final evolution. Angiogenesis in placental tissue begins as early as three weeks post conception and continues throughout pregnancy (24). Meegdes and colleagues found that chorionic villous vascularization in patient with fetal death was significantly lower than in women undergoing legal abortion (10). There is also evidence that pre-eclampsia was caused by disturbances in vascular growth in the fetomaternal unit (25). Intrauterine growth restriction, another reason for perinatal morbidity and mortality induced the developmental disorder of the villous branches and pathological changes in the vasculature of villous in placenta (26). All of this evidence suggest that any disruption in the angiogenesis process in embryo-related structures can lead to insufficiency in the normal development of the embryo/fetus.However, this may not be the only way to disturb the pregnancy process by methenamine and other probable side effects of the drug during pregnancy should befurther investigated.

Methenamine belongs to a class of organic compounds named 1, 3, 5-Triazine. 1, 3, 5-Triazine is a scaffold that has many derivatives. These compounds were extensively studied because of theirdifferent biological effects such as antimicrobial, antiviral, and anti-inflammatory (27-29). Additionally, great attention has been paid to antitumor activity of 1, 3, 5-triazine derivatives (30). It is shown that 2-hydroxy-4, 6-diamino-[1, 3, 5] triazines (a 1,3,5-Triazine derivative) have antiangiogenic property via potent inhibitory effect on VEGF-R2 tyrosine kinase (flk-1/KDR) that functions as the main mediator of VEGF-induced endothelial proliferation, survival, and migration (31). There areother studies in the literature about antiangiogenic effect of 1,3,5-Trazine different derivatives (32, 33). Unlike the other members of 1,3,5-Triazinecompounds,there are no studies that provide evidence on the anticancer properties of methenamine salts, but formaldehyde released from methenamine in acidic PH can inhibit the proliferation and induce apoptosis of cancer cells (34). Antiangiogenic properties of methenamine that revealed inthis study could makethe role of methenamine in treating cancers more prominent.

In the present study, we demonstrated that methenamine at the dose of 150 mg/kg reduced VEGF-A related mRNA. This finding confirmed the fact that methenamine certainly caused an angiogenic disorder. VEGF-A is best known as a key regulator of the angiogenesis process. It is demonstrated that transgenic mice forsingle VEGF-A allele had abnormal disturbed angiogenesis and subsequent embryonic death (35). So, keeping tight control of VEGF-A expression is the important part of normal developmental angiogenesis. The mechanism by which methenamine reduced angiogenesis in thepresent study is unknown, but the amount of VEGF-A was definitely decreasedand could be one of the main reasons for this incident.

In the current study, in silico molecular-modeling assay, using a *Gallus gallus* model of VEGF-A protein, were conducted toprecisely search for the target of methenamine as an antiangiogenic agent. We found a good binding relationship between methenamine and VEGF-A protein through molecular docking. This may provide a link between methenamine exposure and vascular defects.

## 5. Conclusion

In conclusion, chick embryo may be a good preclinical model to assess the adverse effects of drugs during early stages of embryogenesis, in which the evaluation cannot be made in human or other animal trials due to the ethical reasons, and will provide data about probable consequence of drug consumption during gestation to clinicians for recommendation of drug to pregnant women. In this study, we reported the anti-angiogenicproperty of methenamine for the first time in the literature. Additionally, the results reported in this studyallow us to suggest that the use of the methenamine during pregnancy should be considered potentially as vasculo-toxic and because undisturbed angiogenesis is essential for normal fetal growth, caution should be exercised when prescribing itduring pregnancy.

##  Conflict of Interests

There is no conflict of interests.
